# Evaluating the Polarization of Tumor-Associated Macrophages Into M1 and M2 Phenotypes in Human Cancer Tissue: Technicalities and Challenges in Routine Clinical Practice

**DOI:** 10.3389/fonc.2019.01512

**Published:** 2020-01-24

**Authors:** Sharmilla Devi Jayasingam, Marimuthu Citartan, Thean Hock Thang, Anani Aila Mat Zin, Kai Cheen Ang, Ewe Seng Ch'ng

**Affiliations:** ^1^Oncological and Radiological Sciences Cluster, Advanced Medical and Dental Institute (AMDI), Universiti Sains Malaysia, Kepala Batas, Malaysia; ^2^Infectious Disease Cluster, Advanced Medical and Dental Institute (AMDI), Universiti Sains Malaysia, Kepala Batas, Malaysia; ^3^Faculty of Applied Sciences, AIMST University, Kedah, Malaysia; ^4^Department of Pathology, School of Medical Sciences, Universiti Sains Malaysia, Kubang Kerian, Malaysia

**Keywords:** tumor-associated macrophages, M1, M2, immunohistochemistry, CD68, CD163, cancer

## Abstract

Tumor-associated macrophages (TAMs) as immune cells within the tumor microenvironment have gained much interests as basic science regarding their roles in tumor progression unfolds. Better understanding of their polarization into pro-tumoral phenotype to promote tumor growth, tumor angiogenesis, immune evasion, and tumor metastasis has prompted various studies to investigate their clinical significance as a biomarker of predictive and prognostic value across different cancer types. Yet, the methodologies to investigate the polarization phenomena in solid tumor tissue vary. Nonetheless, quantifying the ratio of M1 to M2 TAMs has emerged to be a prevailing parameter to evaluate this polarization phenomena for clinical application. This mini-review focuses on recent studies exploring clinical significance of M1/M2 TAM ratio in human cancer tissue and critically evaluates the technicalities and challenges in quantifying this parameter for routine clinical practice. Immunohistochemistry appears to be the preferred methodology for M1/M2 TAM evaluation as it is readily available in clinical laboratories, albeit with certain limitations. Recommendations are made to standardize the quantification of TAMs for better transition into clinical practice and for better comparison among studies in various populations of patients and cancer types.

## Introduction

Macrophages are a group of differentiated immune cells that are phagocytic in nature and have specific phenotypic characteristics (1). They are a diverse set of immune cells which are polarized by various microenvironmental stimuli to generate a heterogeneous population with different properties and functions. Macrophages are involved in tissue homeostasis, defense mechanisms, and wound healing. They also play a role in various diseases, such as autoimmune disorders, atherosclerosis, and tumorigenesis (1, 2).

Activated macrophages are often classified as pro-inflammatory M1 macrophages or anti-inflammatory M2 macrophages. The M1-like phenotype is induced by toll-like receptor ligands (bacterial lipopolysaccharide) or Th1 cytokines, such as tumor necrosis factor alpha (TNF-α), interferon gamma (IFNγ) and colony stimulating factor 2 (CSF2) (3, 4). M1-like macrophages exert high antigen presenting capacity (5). They also secrete reactive oxygen species (ROS) and cytokines like IL-6, IL-12, IL-23, and TNF-α, associated with microbicidal and pro-inflammatory activities (6). Thus, they are termed as the “fight” macrophages and are associated with good prognosis in cancer context (7, 8).

M2-like macrophages, on the other hand, are polarized by Th2-derived cytokines such as IL4, IL10, IL13, transforming growth factor beta (TGFβ) or prostaglandin E2 (PGE2) (3). They are known as the “repair” or “fix” macrophages as they promote tissue repair *via* immune tolerance and tissue remodeling, debris scavenging and immune modulation (7). When it comes to cancer, M2-like macrophages support angiogenesis by secreting adrenomedullin and vascular epithelial growth factors (VEGFs) and express immunosuppressive molecules such as IL10, programmed death-ligand 1 (PD-L1), and TGFβ, favoring tumor growth (3). They are regarded as “friends” by cancer cells.

It should be noted that the actual polarization state of macrophages is far more complex than the simple binary M1, M2 classification, which serves to define only macrophage functions. In fact, macrophages are highly plastic cells consisting of a spectrum of activation states, with M1 and M2 representing the extremes on each opposing end. Many of the subsets display mixed heterogeneity and some are yet to be discovered or fully characterized. Detailed explanation on macrophage polarization and the mechanisms has been described in a number of recent reviews (1, 3, 9) and thus will not be further elaborated here.

A specific class of macrophages, tumor-associated macrophages (TAMs) are macrophages within the tumor microenvironment (TME). TAMs regulate metastasis by producing growth factors, cytokines, and other molecules. In recent years, researchers have been investigating TAMs as a therapeutic strategy to curb tumor progression and metastasis (9).

## Clinical Significance of Tumor-Associated Macrophages

As a whole, TAMs play a crucial role in cancer progression as supported by many *in-vitro* and *in-vivo* studies (10–12). In parallel to the growing insights into the roles of TAMs, many studies have been conducted to look into the clinical significance of TAMs in solid tumors. Generally, higher densities of TAMs are often observed in more advanced tumor stages as evidenced in esophageal cancer (13), ovarian cancer (14), breast cancer (12), and pancreatic cancer (15). Additionally, the negative impact of TAMs on patients' overall survival was also reflected in esophageal cancer (13), pancreatic cancer (16), breast cancer (17), lung cancer (18), and gastric cancer (19).

## M2 Tumor-Associated Macrophages—The “Aggressor” of Tumor

Higher infiltration of the M2 TAMs is associated with a more aggressive tumor characteristic, reflected by tumor invasion, progression, and metastases. A recent study on non-functional pituitary adenomas (NFPAs) discovered that the cultured M2 macrophages significantly enhanced the proliferation and invasion of the primary NFPA cultures as compared to the cultured M1 macrophages (20). In hepatocellular carcinoma, higher M2 TAMs were strongly correlated with more tumor number and advanced stages (21). Similarly, M2 infiltration in breast cancer was markedly correlated with larger tumor size, advanced stages, and angiogenesis. Polarization toward the M2 phenotype showed strong correlation with the aggressiveness of breast cancer characterized by higher histologic grade, higher Ki-67 proliferating index, estrogen receptor and progesterone receptor negativity (22, 23).

TAMs are also related to resistance toward cancer treatments. M2 macrophages can induce chemoresistance by secreting growth factors and inhibiting cell death signaling pathways in tumor cells, thus sheltering them from the chemotherapy effects (24). Clinical studies have demonstrated that a high number of M2 TAMs in tumor cause chemoresistance and radioprotective effects, leading to therapy failure and poor prognosis in patients (25–27). One study discovered that TAMs skewed toward the M2 phenotype resulted in immuno-tolerance and resistance to anti-Her2/neu therapy in breast cancer. When the anti-Her2/neu therapy was combined with targeted delivery of IL-21, a cytokine that can enhance phagocytosis and protease activity of macrophages, TAM polarization was skewed toward the M1 phenotype instead, thus reversing immunosuppression and resuming tumoricidal activity (28). Hence, higher M2 TAMs were associated with treatment resistance, whereas higher M1 TAMs correlated with improved treatment outcome.

## M1/M2 Ratio as the More Amenable Means of Cancer Prognostication in Clinical Practice

The accentuation on the usage of M1/M2 ratio in prognosticating cancer is supported by the ambiguities concerning the sole densities of TAMs in patients' prognosis (29, 30). For instance, although TAMs in general have negative effect on the gastric patients' prognosis in many studies (19, 31, 32), some studies showed opposing results (33–35). This contradiction is mainly because most studies only involve the total number of macrophages (M1 + M2), instead of evaluating M1 and M2 TAMs separately. In order to resolve these disputes, researches have shown that polarization of TAMs in cancer, more specifically the M1/M2 ratio, is a more biologically relevant indicator to prognose cancer as compared to whole TAM densities (36, 37). This ratio could represent either a positive or a negative impact on tumor growth (14, 29, 38).

A higher M1/M2 ratio in cancer tissue usually signifies a favorable outcome. Petrillo et al. (39) demonstrated that in locally advanced cervical cancers (LACC), a more frequent complete pathological response to chemotherapy (CT) or radiotherapy (RT) was observed in patients with high M1/M2 ratio. On the other hand, polarization of TAMs toward the M2 phenotype as reflected by lower M1/M2 ratio was an independent predictor for poor response to CT or RT and shorter survival in LACC (39). This was supported by other studies involving different cancer types such as ovarian cancer (40), pediatric classical Hodgkin lymphoma (cHL) (41), and multiple myeloma (42).

A lower M1/M2 ratio often indicates poor prognosis in cancer patients, while better prognosis is associated with higher M1/M2 ratio. For instance, in LACC, the tumor microenvironment is mainly skewed toward the M2 phenotype upon diagnosis. Among these patients, those with high M1/M2 ratio exhibited a longer 5-years-disease-free (67.2 vs. 44.3%; *P* = 0.019) and 5 years overall survival (OS) compared to cases with low M1/M2 ratio (69.3 vs. 46.9%, *P* = 0.037) (39). Similarly, in gastric cancer, 27 out of 52 radically resected gastric patients with M1 density above the median had a significantly higher survival rate as compared to those below the median (median survival of 25.6 months vs. 17.6 months, *P* = 0.041). Moreover, 26 patients with M1/M2 ratio above the median showed median OS of 27.2 months as compared to 15.5 months of the patients below the median (37). Hence, the M1 macrophage density and M1/M2 ratio are important factors in predicting patients' survival time.

## Evaluation of M1 and M2 Polarization in Tissue Section: Technicalities

It is evident that the effort to harness TAMs as a biomarker in clinical practice has shifted from simple enumeration of TAM densities to specifically characterizing the vector of polarization phenomena by employing M1/M2 ratio as a surrogate marker (37, 39, 43). In this transition, several technicalities and challenges are worth further scrutinization.

The field of immunology has several advancements in accessing TAMs in tissue biopsies. Among them are flow cytometry, gene expression profiling, and the recent high-throughput single-cell sequencing (44–46). These advancements are no doubt welcomed in the research field as they are more standardized and quantitative in nature. However, when it comes to routine clinical diagnosis where cost, feasibility, and time are restricting factors, the immunohistochemistry is the preferred method. What makes immunohistochemistry distinctive is the potential to assess a cell expression in its own microenvironment (47). Visual details on protein distribution and localization can be procured without the need to destroy the histologic architecture of a tissue (48). Accordingly, studies focusing on TAM characterization in solid clinical samples often adopt immunohistochemistry in their methodologies till date (8, 49, 50). In contrast, methods such as flow cytometry and gene expression are performed *in vitro* and could not reflect the complexity of immune responses observed *in vivo* (51, 52). Hence, when it comes to routine clinical practice, immunohistochemistry of histological section remains the best platform to study TAMs *in situ* and is therefore, the sole emphasis of this review. To expound immunohistochemistry in the context of technicality, there are several important issues to consider.

### Tumor-Associated Macrophages Markers

**Figure 1:**
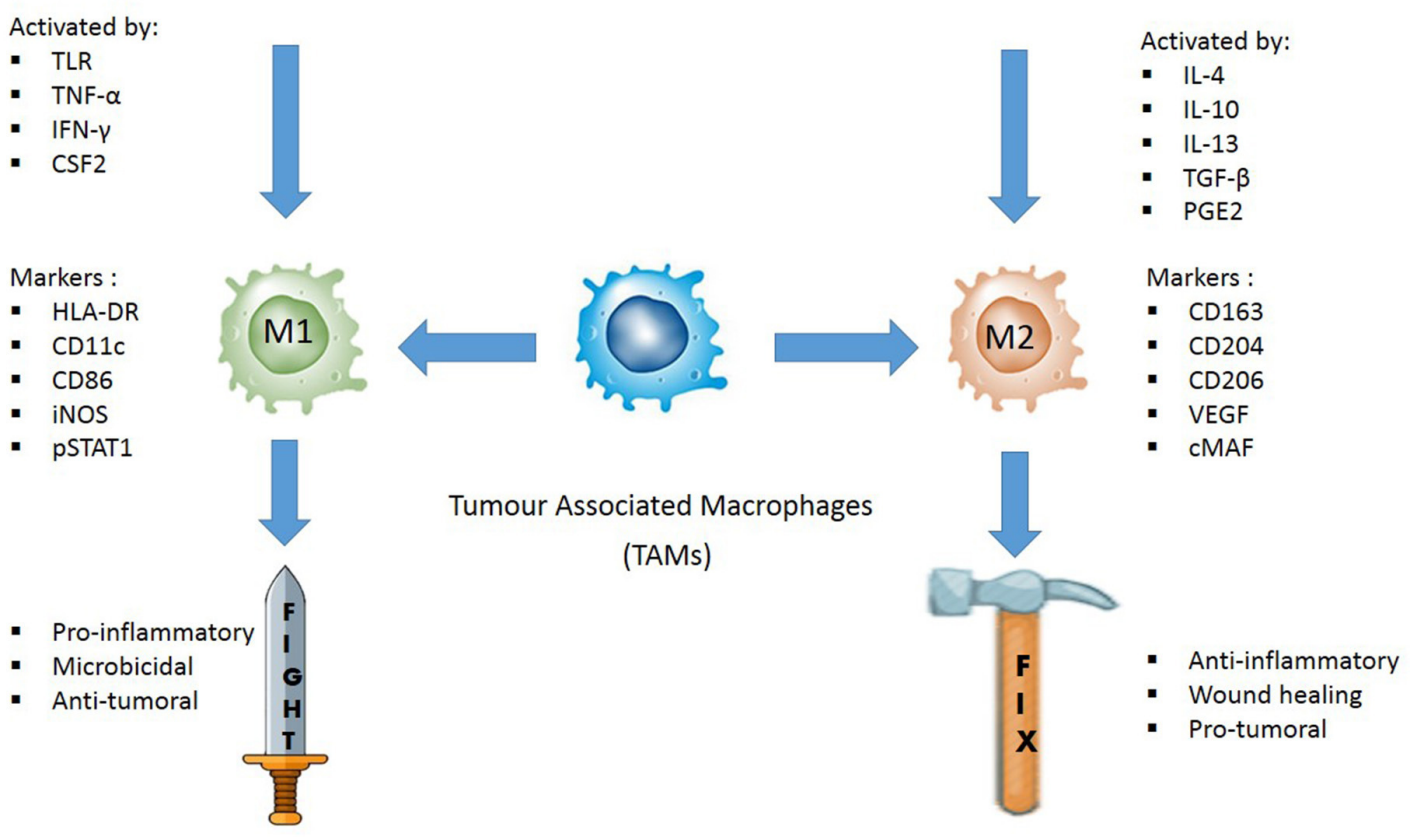
Illustration of how M1 and M2 TAMs are activated, their respective functions, and the common IHC markers used to distinguish the two phenotypes in tissue samples. M1 TAMs are activated by toll-like receptors (TLRs) or Th1 cytokines, such as tumor necrosis factor alpha (TNF-α), interferon gamma (IFNγ), and colony stimulating factor 2 (CSF2). They are pro-inflammatory, microbicidal and have anti-tumoral effect. HLA-DR, CD11c, CD86, iNOS, and pSTAT1 are some of the common IHC markers used to identify M1 TAMs. M2 TAMs are activated by IL4, IL10, IL13, transforming growth factor beta (TGFβ) or prostaglandin E2 (PGE2). They are anti-inflammatory, promotes wound healing, and are pro-tumoral. CD163, CD204, CD206, VEGF, and cMAF are some of the IHC markers used to distinguish M2 TAMs.

Immunohistochemical markers used to identify M1 and M2 TAMs are the cornerstones of TAM evaluation. Immunohistochemistry protocol varies greatly in selecting the best markers to quantify TAMs. Unlike M2, M1 macrophages highly express HLA-DR (53) and inducible nitric oxide synthase (iNOS) in inflamed human tissue (54, 55). Meanwhile, pSTAT1, the phosphorylated form of STAT1, is a transcription factor which promotes M1-associated functions (56). Thus, some common markers for M1 TAMs in human samples are HLA-DR, iNOS, and pSTAT1. In contrast, common markers for M2 TAMs are CD206, CD204, and CD163, attributable to the high expression of the mannose receptor-1 (CD206) and macrophage scavenger receptors (CD204 and CD163) by the M2 TAMs (Figure 1) (57, 58).

Immunohistochemistry also differs in the number of markers employed to identify each population of TAMs. In some studies, only a single marker is used to identify M1 or M2 TAMs, such as CD68 and CD163 (59–61). The single marker-based practice is not encouraged as this affects reproducibility, makes case to case comparison difficult, and the antigens can also be co-expressed by other cells (62). Moreover, dependency on a single marker to evaluate TAMs followed by correlation with clinicopathological findings may yield contradictory results, as reflected by some papers (29, 32, 35). Therefore, the trend has now shifted to double immunohistochemistry staining to better characterize M1 and M2 TAMs. Some of the frequent double stain combinations are CD68/iNOS (63, 64) and CD68/HLA-DR (65, 66) for M1 TAMs and CD68/CD163 for M2 TAMs (23, 65, 66). However, problems may arise if the antigens chosen are co-localized in the same cell compartment. For example, combining CD68/iNOS for M1 TAMs may be difficult to interpret as both markers are localized in the cytoplasm. This poses the risk of causing one color to overshadow the other or resulting in a mixture of colors, complicating visual interpretation (43). The best practice is to select the combination whereby the individual antigens are confined to unique, non-overlapping cellular locations to permit precise visual interpretation.

### Detection Methods

Another important element in immunohistochemistry is choosing the appropriate detection method. Immunohistochemistry is categorized into two main detection methods; immunofluorescence and the chromogenic method. The principles for both methods are similar; the specific antigen-antibody reaction is probed by a labeled antibody to localize the target antigens. This label could either be a fluorochrome or an enzyme/chromogen (67). By convention, the chromogenic method is usually referred as immunohistochemistry (IHC) without specification, while the fluorochrome detection method is known as immunofluorescence (IF).

A scoping search was performed in major databases to assess the application of IHC and IF in quantifying M1 and M2 TAMs in tumorous tissue by retrieving articles from the last 6 years (2014–2019) with the inclusion criterion of human tumor samples. Cell lines and animal models were excluded. Based on the analysis, more than 80% of the papers chose IHC over IF, while IF was mostly performed on cell lines and animal models (Table 1).

**Table 1:** List of articles which evaluated M1 and M2 TAMs in tissue sections from 2014 to 2019.

**References**	**Cancer type**	**Staining type**	**Markers**
Jeong et al. (23)	Invasive breast cancer	IHC	CD68/CD11c—M1 CD 68/CD163—M2
Sousa et al. (68)	Breast cancer	IHC	CD68—pan macrophage HLA-DRα–M1 CD163—M2
Rakaee et al. (66)	Non-small cell lung cancer (NSCLC)	IHC	HLA-DR/CD68—M1 CD163/CD68—M2 CD204/CD68—M2
Almatroodi et al. (63)	Non-small cell lung cancer (NSCLC)	IHC	CD68—pan macrophage iNOS—M1 CD163—M2
Shu et al. (69)	Hepatocellular carcinoma	IHC	CD68—pan macrophage CD11c—M1 CD206—M2
Dong et al. (21)	Hepatocellular carcinoma	IHC	CD68—pan macrophage CD86—M1 CD206—M2
Dumars et al. (64)	Osteosarcoma	IHC	CD68—pan macrophage iNOS—M1 CD163—M2
Lúcio et al. (70)	Squamous cell carcinomas of the lower lip	IHC	CD68—pan macrophage HLA-DR—M1 CD163—M2
Lin et al. (71)	Pulmonary squamous cell carcinomas	IHC	pSTAT1—M1 CD163—M2
Xu et al. (72)	Renal cell carcinoma	IHC	CD68—pan macrophage CD11c—M1 CD206—M2
Kelly et al. (73)	Type 2 endometrial cancer	IHC	CD68—total TAMs CD163—M2 TAMs
Petrillo et al. (39)	Locally Advanced Cervical Cancer (LACC)	IHC	CD68/pSTAT—M1 CD68/cMAF—M2 CD163/pSTAT—M1 CD163/cMAF—M2
Marchesi et al. (65)	Diffuse large B-cell lymphoma	IF	CD68/HLA-DR—M1 CD68/CD163—M2
Yu et al. (74)	Extrahepatic cholangiocarcinoma	IF	CD14—pan macrophage CD14+CD163–:M1 CD14+CD163+ :M2
Lee et al. (75)	Sonic hedgehog (SHH) medulloblastomas	IHC for macrophage recruitment	CD68—pan macrophage CD86—M1 CD163—M2
		IF to confirm localization	CD86—M1 CD163—M2
Zhang et al. (40)	Ovarian cancer	IHC for macrophage recruitment	CD68—pan macrophage
		IF to confirm localization	CD68—pan macrophage iNOS—M1 HLA-DR—M1 CD163—M2 VEGF—M2
Mori et al. (76)	Oral squamous cell carcinoma	IHC for macrophage recruitment	CD68—pan macrophage CD80—M1 CD163—M2
		IF to confirm localization	STAT1/pSTAT1—M1 CD163—M2

In routine clinical settings, IHC is mainly preferred over IF due to the convenience of the formalin-fixed paraffin embedded (FFPE) tissue, i.e., huge amounts of archived clinical specimens owing to the simplicity and low cost (77). Furthermore, a fluorescence microscope is not required; a simple light microscope would suffice to view the IHC stain. The stained samples can also be stored for a long time as the chromogenic stains are more resistant to photobleaching. Hence, IHC using FFPE tissue blocks has high importance in retrospective studies.

Hitherto, IF is not routinely used in clinical settings due to several limitations. Firstly, IF usually requires a fresh frozen tissue where the proteins are preserved in their native state. Fixation with formalin for instance could be harsh on the tissue, blocking the epitopes of the target antigen or altering them. Blockade of the epitopes impairs their recognition by antibodies, resulting in poor signal production. There is also the risk of autofluorescence in IF if FFPE tissue was used (48). Besides, frozen tissue could prove to be costly, as it requires specialized equipment to maintain the samples (78). Furthermore, IF necessitates a designated, cool, and darkened room for the slides to be read as IF is highly susceptible to photo bleaching. This could be inconvenient in cases where re-reading of the slides is necessary.

### Localization of Tumor-Associated Macrophages: Tumor Stroma vs. Tumor Nests

One main reason for the discrepancy in TAMs effect on clinicopathological parameters is the location of TAM infiltration. TAMs in different tumor compartments may have a different implication on cancer prognosis. For example, in breast cancer studies, infiltration of M2 TAMs in tumor stroma instead of tumor nests was associated with larger tumor size, higher histological grade, higher 5-year recurrence, and 5-year breast cancer mortality, in addition to being an independent predictor of recurrence-free survival (RFS) and OS (23, 79). Similarly, in esophageal carcinoma, infiltration of M2 TAMs in tumor stroma was strongly associated with more malignant characteristics such as metastasis and clinical stage progression. This highlights the importance of considering the localization of TAMs in addition to their number as a prognostic marker.

### Quantification of Tumor-Associated Macrophages

Most researches often employ the “hotspot” quantitative method to evaluate TAMs. However, this method varies in the number of fields selected for TAM evaluation and the counting method performed. In some studies, 10 “hot” fields were chosen to quantify TAMs before obtaining an average, while others settled with three or five “hot” fields. As for counting TAMs, certain papers applied software such as ImageScope and ImageJ Cell, while others consulted two pathologists with vast experience to quantify TAMs manually (23, 39, 69, 70, 75).

## Challenges and Strategies to Address the Limitations in the Evaluation of M1 and M2 Polarization

### Poor Standardization of Tumor-Associated Macrophage Quantification

Regardless of IHC or IF, a persisting disadvantage of the immunohistochemistry is the obvious lack of standardization in TAM assessment. Discrepancies in TAM assessment stem from the variation in tissue collection, tissue fixation, tissue thickness, assessment criteria, staining process, and image analysis. One prominent example is the variation in selecting the best markers to quantify TAMs. Different markers are used across different studies as reflected in Table 1. Inconsistencies in TAM assessment are more evident between laboratories. Thus, reproducibility is significantly low when the same specimen is evaluated by different laboratories (44). Although concerns have been raised regarding low reproducibility of the immunohistochemistry for the past decades, till date, only minimal efforts have been taken to improve its quality (44, 47, 80, 81). Absolute standardization in immunohistochemistry protocol is difficult; however, small steps taken gradually will eventually lead to a more robust and global operating procedures. For starters, all laboratories should adhere to a fixed section thickness for TAM assessment. In quantifying M1 and M2 TAMs, all researchers/pathologists should reach a consensus of using 10 “hot” spots for a better reflection of the TAM number.

### Lack of Robust Marker Confounds the Quantification of Tumor-Associated Macrophages

Another concerning matter is the need for a highly specific yet flexible marker. For instance, antibodies for CD68, the pan macrophage marker, are not specific for cells of the monocyte/macrophage system besides being immunohistochemically detectable in a variety of other cell types (82). iNOS, a popular M1 macrophage marker, is co-expressed by endothelial cells and arterial wall smooth muscle cells (37). Moreover, there are controversies regarding its use due to the difference in expressions in murine and human macrophages (83, 84). CD163, a M2 macrophage marker, is also expressed in some dendritic and endothelial cells (60). This could result in inaccurate M1 and M2 TAM counts. One way to minimize false positive count is to amalgamate quantification of TAMs with the visualization of cell morphology.

The complex nature of macrophage heterogeneity undermines many clinical studies. Although the application of M1/M2 TAM ratio in tumorous tissue has provided meaningful insights into cancer prognosis, it does not reflect the exact functions of these cells; macrophages *in vivo* exist in a continuum of functional states and may display both M1 and M2 markers, making it difficult to interpret their impact (85). To address this complication, methods like gene expression, cytokine profiling, or miRNA expression can be used to verify their polarization and functions. Nevertheless, the best solution would be the discovery of novel markers which truly reflect TAM heterogeneity. There will be higher possibility for this discovery when the conundrum of macrophage classification is resolved.

### Variation in Interpretation

In immunohistochemistry, it is difficult to address the variability in human perception for its visual analysis (48). Hence, quantification of M1 and M2 TAMs in a tissue section is prone to bias and inter-observation (5, 86). Digital image analysis has been introduced to reduce subjective/human error. With this technology, the M1 and M2 TAM count could be automated, ensuring precise and accurate result each time. However, this application could wrongly misinterpret cells such as dendritic and endothelial cells as TAMs. Therefore, a software which can integrate both the automated count and cell visualization to ascertain cell type can yield more reliable and specific TAM count. Although not meant for TAMs, softwares have been developed to enhance the microscopic image analysis. The ImmunoRatio software for example, segments immunostained and hematoxylin-stained cellular areas from the user-submitted image and calculates the labeling index for ER, PR, and Ki-67 in breast cancer (87). With some modifications, it is conceivable that this type of software can be applied for quantification of M1/M2 ratio in solid tumor in future.

## Conclusion

The ultimate goal of every pathologist or researcher is to be able to evaluate M1 and M2 TAMs in the most specific and standardized manner for a clear-cut determination of the TAM polarization. For IHC to be considered as a “top-rate” biomarker, optimization of all aspects of IHC is critically needed to develop a universal protocol which is reliable and reproducible for the determination of the polarization of TAMs. This mini review provides caveats as well as insights to better handle this important progressive aspect of solid tumor biology.

## Author Contributions

EC and SJ conceived and prepared the manuscript. MC, TT, AM, and KA contributed to essential input and manuscript editing.

### Conflict of Interest

The authors declare that the research was conducted in the absence of any commercial or financial relationships that could be construed as a potential conflict of interest. The handling Editor declared a shared affiliation, though no other collaboration, with the authors at time of review.
